# Immune Checkpoint Inhibitors for Child-Pugh Class B Advanced Hepatocellular Carcinoma: A Systematic Review and Meta-Analysis

**DOI:** 10.1001/jamaoncol.2023.3284

**Published:** 2023-08-24

**Authors:** Enrui Xie, Yee Hui Yeo, Bernhard Scheiner, Yue Zhang, Atsushi Hiraoka, Xinxing Tantai, Petros Fessas, Tiago de Castro, Antonio D’Alessio, Claudia Angela Maria Fulgenzi, Shuo Xu, Hong-Ming Tsai, Swetha Kambhampati, Wenjun Wang, Bridget P. Keenan, Xu Gao, Zixuan Xing, Matthias Pinter, Yih-Jyh Lin, Zhanjun Guo, Arndt Vogel, Takaaki Tanaka, Hsin-Yu Kuo, Robin K. Kelley, Masatoshi Kudo, Ju Dong Yang, David J. Pinato, Fanpu Ji

**Affiliations:** 1 Department of Infectious Diseases, The Second Affiliated Hospital of Xi’an Jiaotong University, Xi’an, China; 2 Karsh Division of Gastroenterology and Hepatology, Cedars-Sinai Medical Center, Los Angeles, California; 3 Department of Surgery and Cancer, Imperial College London, United Kingdom; 4 Division of Gastroenterology and Hepatology, Department of Internal Medicine III, Medical University of Vienna, Austria; 5 The Eighth Hospital of Xi’an City, Xi’an Jiaotong University, Shaanxi, China; 6 Gastroenterology Center, Ehime Prefectural Central Hospital, Matsuyama, Japan; 7 Department of Gastroenterology, The Second Affiliated Hospital of Xi’an Jiaotong University, Xi’an, China; 8 Department of Gastroenterology, Hepatology, Infectious Diseases and Endocrinology, Hannover Medical School, Hannover, Germany; 9 Department of Rheumatology and Immunology, The Fourth Hospital of Hebei Medical University, Shijiazhuang, China; 10 Department of Diagnostic Radiology, National Cheng Kung University Hospital, College of Medicine, National Cheng Kung University, Tainan, Taiwan; 11 Department of Hematology and Hematopoietic Stem Cell Transplantation, City of Hope National Medical Center, Duarte, California; 12 Division of Hematology/Oncology, Department of Medicine, Helen Diller Family Comprehensive Cancer Center, University of California, San Francisco, San Francisco; 13 Department of Surgery, National Cheng Kung University Hospital, College of Medicine, National Cheng Kung University, Tainan, Taiwan; 14 Department of Internal Medicine, National Cheng Kung University Hospital, College of Medicine, National Cheng Kung University, Tainan, Taiwan; 15 Institute of Clinical Medicine, College of Medicine, National Cheng Kung University, Tainan, Taiwan; 16 Department of Gastroenterology and Hepatology, Kindai University Faculty of Medicine, Osaka, Japan; 17 Samuel Oschin Comprehensive Cancer Institute, Cedars-Sinai Medical Center, Los Angeles, California; 18 Division of Oncology, Department of Translational Medicine, University of Piemonte Orientale “A Avogadro,” Novara, Italy; 19 National and Local Joint Engineering Research Center of Biodiagnosis and Biotherapy, The Second Affiliated Hospital of Xi’an Jiaotong University, Xi’an, China; 20 Shaanxi Provincial Clinical Medical Research Center of Infectious Diseases, Xi’an, China; 21 Key Laboratory of Surgical Critical Care and Life Support (Xi’an Jiaotong University), Ministry of Education, Xi’an, China

## Abstract

**Importance:**

Immune checkpoint inhibitors (ICIs) are increasingly used in patients with advanced hepatocellular carcinoma (HCC). However, data on ICI therapy in patients with advanced HCC and impaired liver function are scarce.

**Objective:**

To conduct a systematic review and meta-analysis to determine the efficacy and safety of ICI treatment for advanced HCC with Child-Pugh B liver function.

**Data Sources:**

PubMed, Embase, Web of Science, and Cochrane Library were searched for relevant studies from inception through June 15, 2022.

**Study Selection:**

Randomized clinical trials, cohort studies, or single-group studies that investigated the efficacy or safety of ICI therapy for Child-Pugh B advanced HCC were included.

**Data Extraction and Synthesis:**

The Preferred Reporting Items for Systematic Reviews and Meta-Analysis guideline was followed to extract data. A random-effects model was adopted if the heterogeneity was significant (*I*^2^ > 50%); otherwise, a fixed-effect model was used.

**Main Outcomes and Measures:**

The objective response rate (ORR) and overall survival (OS) were considered to be the primary efficacy outcomes of ICI treatment for Child-Pugh B advanced HCC, and the incidence of treatment-related adverse events (trAEs) was set as the primary measure for the safety outcome.

**Results:**

A total of 22 studies including 699 patients with Child-Pugh B and 2114 with Child-Pugh A advanced HCC comprised the analytic sample (median age range, 53-73 years). Upon pooled analysis, patients treated with ICIs in the Child-Pugh B group had an ORR of 14% (95% CI, 11%-17%) and disease control rate (DCR) of 46% (95% CI, 36%-56%), with a median OS of 5.49 (95% CI, 3.57-7.42) months and median progression-free survival of 2.68 (95% CI, 1.85-3.52) months. The rate of any grade trAEs in the Child-Pugh B group was 40% (95% CI, 34%-47%) and of grade 3 or higher trAEs was 12% (95% CI, 6%-23%). Compared with the Child-Pugh A group, the ORR (odds ratio, 0.59; 95% CI, 0.43-0.81; *P* < .001) and DCR (odds ratio, 0.64; 95% CI, 0.50-0.81; *P* < .001) were lower in the Child-Pugh B group. Child-Pugh B was independently associated with worse OS in patients with advanced HCC treated with ICIs (hazard ratio, 2.72 [95% CI, 2.34-3.16]; adjusted hazard ratio, 2.33 [95% CI, 1.81-2.99]). However, ICIs were not associated with increased trAEs in the Child-Pugh B group.

**Conclusions and Relevance:**

The findings of this systematic review and meta-analysis suggest that although the safety of ICI treatment was comparable between patients with HCC with vs without advanced liver disease and the treatment resulted in a significant number of radiologic responses, survival outcomes are still inferior in patients with worse liver function. More study is needed to determine the effectiveness of ICI treatment in this population.

## Key Points

**Question:**

What are the efficacy and tolerability of immune checkpoint inhibitors (ICIs) for patients with advanced hepatocellular carcinoma (HCC) with Child-Pugh B liver function?

**Findings:**

In this systematic review and meta-analysis of 22 studies involving 699 patients with Child-Pugh B and 2114 patients with Child-Pugh A advanced HCC, ICI therapy in the Child-Pugh B group appeared to be safe and showed a significant number of radiologic responses, but survival outcomes were inferior compared with the Child-Pugh A group.

**Meaning:**

These findings suggest that although the overall prognosis is still poor for patients with HCC, a proportion of patients with Child-Pugh B liver function might benefit from ICI therapy.

## Teaser

This systematic review and meta-analysis evaluates the efficacy and safety of immune checkpoint inhibitors in the treatment of advanced hepatocellular carcinoma with Child-Pugh class B liver function.

## Introduction

Liver cancer is the third leading cause of cancer-related deaths, is sixth in global incidence for cancers,^1^ and has been increasing in many regions of the world.^2^ Hepatocellular carcinoma (HCC) accounts for approximately 90% of primary liver cancer.^3^ Due to late-stage diagnosis, most patients with HCC are not eligible for surgical or locoregional treatment, making systemic treatment the mainstay treatment option.^4^ However, varying degrees of hepatic dysfunction resulting from underlying liver disease are associated with the prognosis and efficacy of systemic treatment.^5^

Sorafenib was the standard first-line treatment for advanced HCC,^6^ but its therapeutic effect in patients with impaired liver function is poor. Prior studies have shown that overall survival (OS) is significantly lower in patients with Child-Pugh class B vs Child-Pugh class A liver function.^7,8,9,10^ Hence, treatment should be individualized for patients with Child-Pugh B cirrhosis.^11^

The emergence of immune checkpoint inhibitors (ICIs) has shown promising results in trials of advanced HCC.^12,13^ Compared with sorafenib, nivolumab treatment has been associated with improved OS and better tolerability in patients with Child-Pugh B HCC .^14^ Subgroup analysis of cohort 5 of the prospective CheckMate 040 study, which included 49 patients with Child-Pugh B cirrhosis, showed similar radiologic response and safety but worse survival compared with patients with Child-Pugh A cirrhosis. Some patients with Child-Pugh B cirrhosis, particularly responders, even showed an improvement in liver function.^15^

Despite the considerable knowledge gap, the majority of patients participating in clinical trials assessing ICI treatment typically are required to have Child-Pugh A liver function, although many patients with advanced HCC actually present with impaired liver function and higher Child-Pugh classes. Therefore, we performed a systematic review and meta-analysis with original data collected from the primary studies to evaluate the efficacy and safety of ICI therapy in patients with Child-Pugh B advanced HCC.

## Methods

### Search Strategy and Selection Criteria

This systematic review and meta-analysis was performed in accordance with the Preferred Reporting Items for Systematic Reviews and Meta-Analyses (PRISMA) guideline^16^ (eAppendix 3 in Supplement 1), and its protocol was registered with PROSPERO (CRD42022379407). We searched PubMed, Embase, Web of Science, and Cochrane Library from inception to June 15, 2022, without language restrictions. References in eligible articles were also searched when necessary. The search strategy is detailed in eAppendix 1 in Supplement 1. Two reviewers (E.X. and Y.Z.) independently completed the title and abstract screening for eligibility using a preplanned list of inclusion and exclusion criteria (eAppendix 2 in Supplement 1), with discrepancies resolved by consensus or discussion with either Y.H.Y. or F.J. The institutional review board of the Second Affiliated Hospital of Xi’an Jiaotong University waived the need for review and informed consent because the study did not involve direct interaction with human participants or the collection of new data.

### Data Extraction and Quality Assessment

Two reviewers (E.X. and Y.Z.) independently extracted data from each study, including the first author’s name, study characteristics, therapeutic method, patient demographic characteristics, HCC etiology, liver function, Barcelona Clinic Liver Cancer stage, duration of follow-up, and relevant outcomes. We collected but did not analyze data on sex due to unavailability of detailed subgroup data. Data on race and ethnicity were not collected due to heterogeneity in data availability and classification methods. When necessary, original authors were contacted to supplement missing or unclear information. The 2 reviewers independently assessed study quality, resolving disagreements through consensus or consultation with authors Y.H.Y. or F.J. The Newcastle-Ottawa Scale was used for observational studies,^17^ designating those scoring 7 or higher as high quality and those scoring 4 to 6 as fair quality. For single-group studies, an Institute of Health Economics (IHE) case series studies quality appraisal tool was used,^18^ with those meeting 70% or more of the 20 criteria deemed acceptable.

### Statistical Analysis

The primary outcomes of this meta-analysis were objective response rate (ORR), OS, and treatment-related adverse events (trAEs) among patients with Child-Pugh B advanced HCC treated with ICIs and how these compared with patients with Child-Pugh A advanced HCC. The secondary outcomes were disease control rate (DCR), progression-free survival (PFS), and immunotherapy-related adverse events (irAEs) in patients with Child-Pugh B vs those with Child-Pugh A. For dichotomous data, we calculated odds ratios (ORs) and 95% CIs as summary statistics. Hazard ratios (HRs) were used as a measure of the prognostic value to analyze PFS and OS. Unadjusted or adjusted HRs and 95% CIs were extracted from studies and then pooled separately. Considering that some studies did not report HRs and 95% CIs, the methods described by Liu et al^19^ were used to obtain the survival data from the reported Kaplan-Meier (K-M) curves. In the single-group study that only included patients with Child-Pugh B HCC, outcomes including ORR, DCR, PFS, OS, and incidence of trAEs or irAEs were pooled with the same variable derived from the randomized clinical trials or cohort studies with both Child-Pugh A and B groups. A generalized linear mixed model was used for the meta-analysis of single proportions. Heterogeneity across included studies was assessed using the Cochran *Q* and *I*^2^ statistics. If the *I*^2^ statistic was less than 50% or the *P* value greater than .10, the heterogeneity was considered to be low, and the fixed-effect model was applied. Otherwise, the random-effects model was applied. We also performed meta-regression analyses to explore potential sources of heterogeneity among the studies. The following study characteristics were included for analytic purposes: year of publication, study design (retrospective or prospective), sample size, proportion of nonviral etiology, proportion of Barcelona Clinic Liver Cancer C/D stage, median follow-up, ICI regimens (monotherapy, combined with targeted therapy, with or without other therapy), and median age. Publication bias was evaluated using the Begg test and funnel plot. The survival, ggplot2, IPDfromKM, and meta packages in R, version 4.1.3 software (R Project for Statistical Computing) were used for statistical analyses. The threshold of statistical significance was set at a 2-sided *P* < .05.

## Results

### Study Selection and Characteristics

Our search yielded 11 200 articles, with 22 studies including 699 patients with Child-Pugh B and 2114 with Child-Pugh A advanced HCC comprising the analytic sample^14,15,20,21,22,23,24,25,26,27,28,29,30,31,32,33,34,35,36,37,38,39^ (eFigure 1 in Supplement 1). Additional data were acquired from 7 studies (220 patients with Child-Pugh B and 787 with Child-Pugh A advanced HCC) through author correspondence.^23,24,26,27,30,33,35^

Study characteristics are shown in eTable 1 in Supplement 1. In summary, 19 of the 22 studies were retrospective,^14,20,21,22,23,24,25,27,28,29,30,31,32,33,34,35,36,37,38^ 3 were prospective,^15,26,39^ 6 evaluated nivolumab,^14,15,21,22,23,26^ 4 evaluated nivolumab and pembrolizumab,^24,25,27,28^ 1 evaluated camrelizumab,^29^ 1 evaluated pembrolizumab,^39^ 5 evaluated atezolizumab,^31,32,33,34,35^ and the remaining 5 did not describe detailed agents.^20,30,36,37,38^ The median patient age ranged from 53 to 73 years among included studies. Median follow-up time ranged from 3.3 to 30.0 months. All studies were rated as high quality based on a Newcastle-Ottawa Scale score greater than 7 or meeting more than 14 IHE criteria (eTables 2 and 3 in Supplement 1).

### Evaluation of Response to Treatment

**Figure 1.**
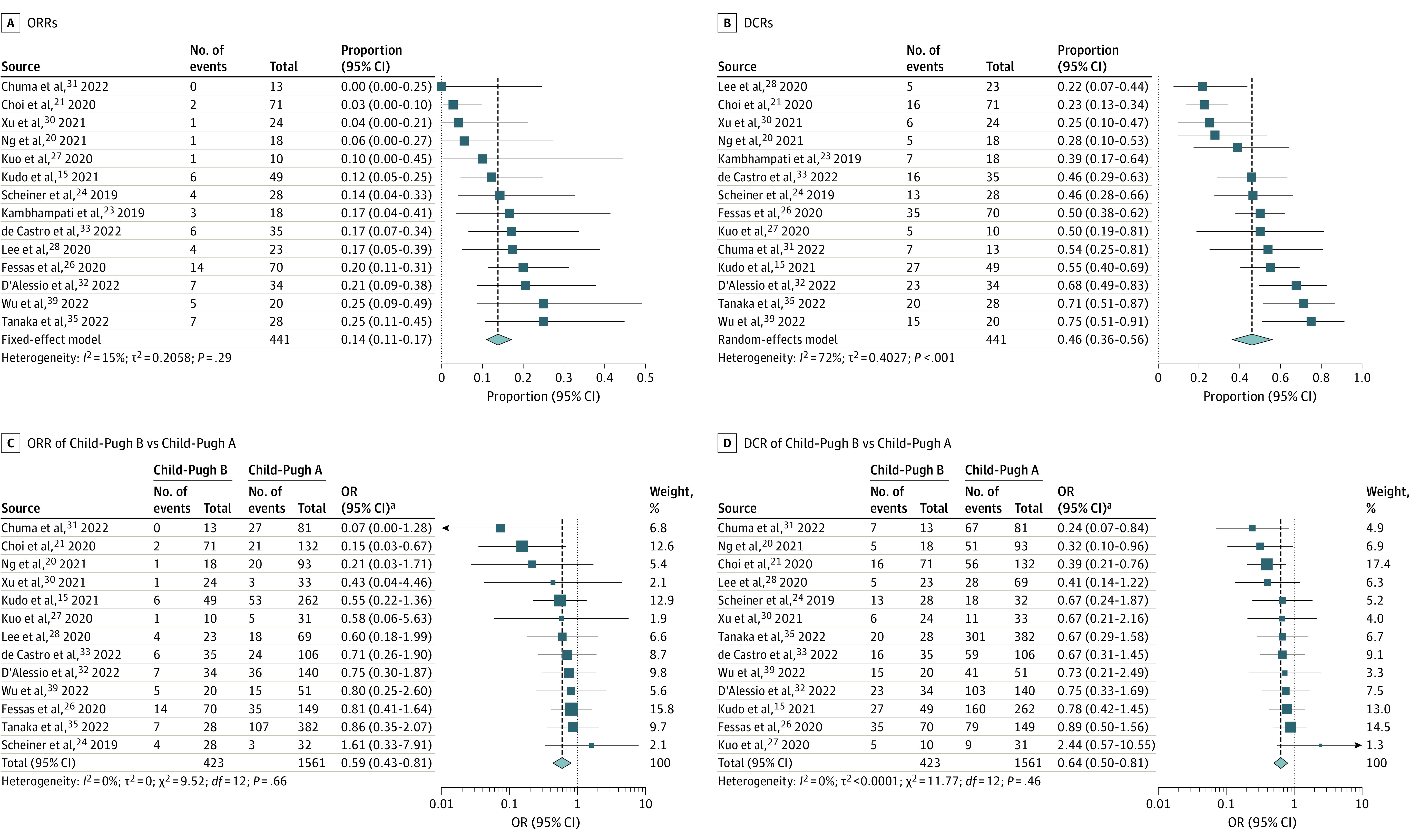
Immune Checkpoint Inhibitor Treatment in Patients With Advanced Hepatocellular Carcinoma (HCC) and Child-Pugh B Liver Function Squares indicate estimates; size of squares, study weights; whiskers, 95% CIs; diamonds, mean estimates; DCR, disease control rate; OR, odds ratio; and ORR, objective response rate. ^a^Mantel-Haenszel test, fixed effects.

There were 14 studies that reported ORR and DCR data for patients with Child-Pugh B advanced HCC treated with ICIs.^15,20,21,23,24,26,27,28,30,31,32,33,35,39^ The pooled ORR was 14% (95% CI, 11%-17%), and the pooled DCR was 46% (95% CI, 36%-56%) (Figure 1A and B). Comparison of pooled cohorts of patients with Child-Pugh B vs Child-Pugh A liver function showed that the ORR and DCR of the Child-Pugh B group were lower, with pooled ORs of 0.59 (95% CI, 0.43-0.81; *P* < .001) and 0.64 (95% CI, 0.50-0.81; *P* < .001), respectively (Figure 1C and D).

Five studies evaluated ICI monotherapy,^15,21,23,24,26^ and 5 investigated ICI combinations with targeted therapy.^31,32,33,35,39^ Upon single-group meta-analysis, subgroup analyses based on ICI regimens showed that the ORR of the Child-Pugh B group was 12% (95% CI, 6%-20%) for ICI monotherapy and 19% (95% CI, 13%-27%) for ICIs combined with targeted therapy. The DCR of the Child-Pugh B group was 42% (95% CI, 31%-54%) for ICI monotherapy and 62% (95% CI, 54%-70%) for ICIs combined with targeted therapy (eFigure 2 in Supplement 1). Significant differences in ORR and DCR between the Child-Pugh B and Child-Pugh A groups were also observed for ICI monotherapy, with ORs of 0.58 (95% CI, 0.36-0.94; *P* = .03) and 0.67 (95% CI, 0.48-0.93; *P* = .02), respectively. For ICIs combined with targeted therapy, the ORR was not significant between the 2 groups, while the DCR was significantly lower in the Child-Pugh B group (eFigure 3 in Supplement 1). Furthermore, we performed a subgroup analysis based on Child-Turcotte-Pugh (CTP) scores (CTP8/9 vs CTP7) in the Child-Pugh B group.^23,24,26,27,33,35^ There were no significant differences in ORR (OR, 0.79; 95% CI, 0.38-1.63; *P* = .52) and DCR (OR, 0.67; 95% CI, 0.37-1.20; *P* = .18) (eFigure 4A and B in Supplement 1).

### Evaluation of Survival Outcomes

**Figure 2.**
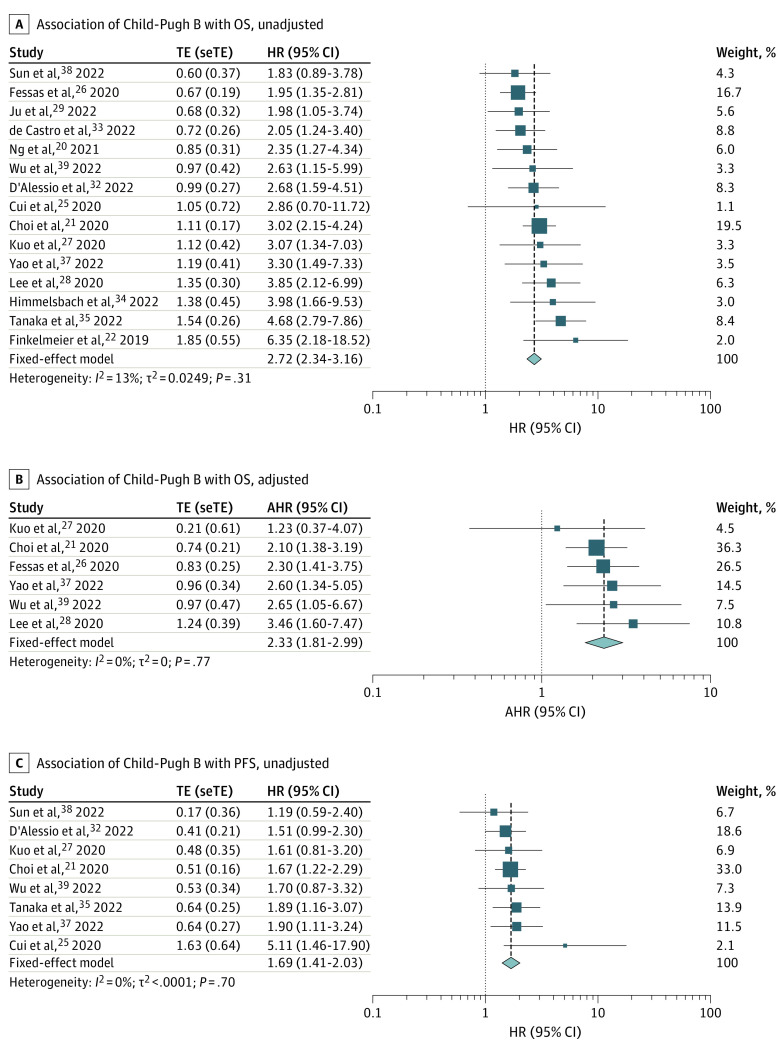
Estimated Overall Survival (OS) and Progression-Free Survival (PFS) in Patients With Advanced Hepatocellular Carcinoma and Child-Pugh B vs A Liver Function Squares indicate estimates; size of squares, study weights; whiskers, 95% CIs; diamonds, mean estimates; AHR, adjusted hazard ratio; HR, hazard ratio; TE, estimate of treatment effect; and seTE, standard error of treatment effect estimate.

**Figure 3.**
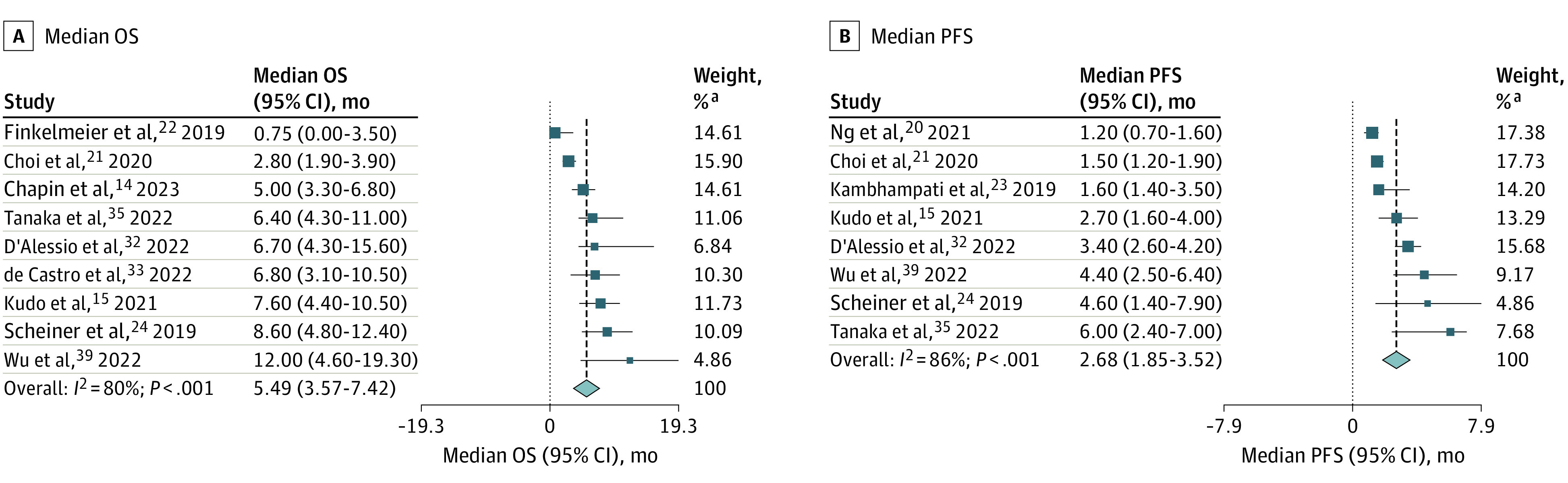
Association of Immune Checkpoint Inhibitors With Median Overall Survival (OS) and Progression-Free Survival (PFS) in Patients With Hepatocellular Carcinoma and Child-Pugh B Liver Function Squares indicate estimates; size of squares, study weights; whiskers, 95% CIs; and diamonds, mean estimates. ^a^Weights are from random-effects analysis.

For the 4 trials not reporting HRs of patients with Child-Pugh B vs Child-Pugh A advanced HCC but providing K-M curves,^22,32,34,35^ time-to-event outcomes were reconstructed from the K-M curves. A side-by-side comparison of the original curves and the reconstructed curves showed a close match to the original K-M curves on visual inspection and comparisons of the number-at-risk tables (eFigure 5 in Supplement 1). We next used the reconstructed individual patient data to calculate the HR. From 15 studies that provided data on univariable analysis of OS,^20,21,22,25,26,27,28,29,32,33,34,35,37,38,39^ Child-Pugh B was associated with an increased mortality risk compared with Child-Pugh A, with a pooled HR of 2.72 (95% CI, 2.34-3.16; *P* < .001) and low heterogeneity (*I*^2^ = 13%; *P* = .31) (Figure 2A). According to data from multivariable analysis reported by 6 studies,^21,26,27,28,37,39^ Child-Pugh B remained associated with increased mortality, with a pooled adjusted HR of 2.33 (95% CI, 1.81-2.99; *P* < .001) and with very low heterogeneity (*I*^2^ = 0%; *P* = .77) (Figure 2B). In addition, 8 studies provided data on univariable analysis of PFS.^21,25,27,32,35,37,38,39^ The pooled unadjusted HR was 1.69 (95% CI, 1.41-2.03; *P* < .001) (Figure 2C). An adjusted HR for PFS was unavailable, as only 2 studies reported the adjusted HR.^21,37^ The pooled median OS and median PFS following ICI treatment for Child-Pugh B advanced HCC were 5.49 months (95% CI, 3.57-7.42 months) and 2.68 months (95% CI, 1.85-3.52 months), respectively (Figure 3).

Because of the limited number of studies, we only performed subgroup analyses of unadjusted HRs of OS based on ICI regimens. Regardless of ICI regimens, patients with Child-Pugh B advanced HCC had a poorer prognosis than those with Child-Pugh A (eFigure 6 in Supplement 1). Notably, we did not observe any differences in OS (HR, 1.08; 95% CI, 0.73-1.60; *P* = .69) and PFS (HR, 1.19; 95% CI, 0.66-2.13; *P* = .57) between patients with CTP8/9 and CTP7 (eFigure 4D and E in Supplement 1).

### Adverse Events

In the Child-Pugh B group, the incidence rate of any grade trAEs was 40% (95% CI, 34%-47%); grade 3 or higher trAEs, 12% (95% CI, 6%-23%); any grade irAEs, 31% (95% CI, 19%-46%); and grade 3 or higher irAEs, 7% (95% CI, 4%-13%). Importantly, the rate of any grade trAEs or grade 3 or higher trAEs was not increased in the Child-Pugh B group vs the Child-Pugh A group. Similarly, the incidence of trAEs was comparable between patients with CTP8/9 and CTP7 (eFigure 4C in Supplement 1). Notably, the risk of irAEs was even lower in the Child-Pugh B group than the Child-Pugh A group, with a pooled OR of 0.49 (95% CI, 0.32-0.73; *P* < .001). Further details on trAEs and irAEs and the number of included studies and patients are shown in the Table.

**Table.** Pooled Incidence of Adverse Events

Group	Child-Pugh A	Child-Pugh B	Child-Pugh B vs A
Any grade trAEs			
IR (95% CI), % or OR (95% CI)^a^	47 (24-71)	40 (34-47)	0.75 (0.31-1.77)
Heterogeneity	*I*^2^ = 90%, *P* < .001	*I*^2^ = 38%, *P* = .16	*I*^2^ = 81%, *P* < .001
No. of studies	5^15,20,24,26,39^	6^15,20,23,24,26,39^	5^15,20,24,26,39^
No. of patients	596	211	789
Grade ≥3 trAEs			
IR (95% CI), % or OR (95% CI)^a^	11 (6-19)	12 (6-23)	1.01 (0.62-1.64)
Heterogeneity	*I*^2^ = 85%, *P* < .001	*I*^2^ = 68%, *P* = .008	*I*^2^ = 0%, *P* = .91
No. of studies	5^15,20,21,24,26^	6^15,20,21,23,24,26^	5^15,20,21,24,26^
No. of patients	677	262	921
Any grade irAEs			
IR (95% CI), % or OR (95% CI)^a^	45 (32-58)	31 (19-46)	0.49 (0.32-0.73)
Heterogeneity	*I*^2^ = 93%, *P* < .001	*I*^2^ = 74%, *P* = .004	*I*^2^ = 0%, *P* = .88
No. of studies	4^13,15,32,36^	5^15,23,30,32,36^	4^15,30,32,36^
No. of patients	580	176	738
Grade ≥3 irAEs			
IR (95% CI), % or OR (95% CI)^a^	12 (8-17)	7 (4-13)	NA
Heterogeneity	*I*^2^ = 70%, *P* = .07	*I*^2^ = 31%, *P* = .24	
No. of studies	2^15,32^	3^15,23,32^	
No. of patients	416	115	

Abbreviations: IR, incidence rate; irAE, immunotherapy-related adverse event; NA, not available; OR; odds ratio; trAE, treatment-related adverse event.

^a^ Incidence rates are reported for the individual Child-Pugh A and Child-Pugh B groups. Odds ratios are reported for Child-Pugh B vs Child-Pugh A.

### Meta-Regression Analyses

We conducted meta-regression analyses on primary outcomes with high heterogeneity (eTables 4 and 5 in Supplement 1). We found no significant associations among the pooled incidence of trAEs in patients with Child-Pugh B vs Child-Pugh A advanced HCC, pooled median OS in the Child-Pugh B group, and the variables of interest.

### Sensitivity Analysis and Publication Bias

To assess whether any single study had a dominant effect on the meta-analysis, we excluded 1 study at a time and analyzed its effect on the main summary estimate. In this analysis, no single study significantly affected the outcome or the heterogeneity (eFigure 7 in Supplement 1). In addition, there was no evidence of publication bias based on the Begg test (eFigure 8 in Supplement 1).

## Discussion

In this systematic review and meta-analysis of data from 22 studies^14,15,20,21,22,23,24,25,26,27,28,29,30,31,32,33,34,35,36,37,38,39^ including 699 patients with Child-Pugh B and 2114 with Child-Pugh A advanced HCC, we found very low heterogeneity across the majority of primary outcome measures analyzed. We obtained original data from 7 main studies^23,24,26,27,30,33,35^ and found that the ORR and DCR of patients with Child-Pugh B advanced HCC treated with ICIs were 14% and 46%, respectively, with a median OS of 5.49 months and a median PFS of 2.68 months. The incidence rate of any grade trAEs was 40%, including 12% for grade 3 or higher. Furthermore, the incidence rate of any grade irAEs was 31%, including 7% for grade 3 or higher. Although patients with Child-Pugh B HCC achieved lower ORRs and DCRs and had shorter OS and PFS than those with Child-Pugh A HCC, ICI treatment was not associated with a significantly higher risk of any or severe trAEs, and a subset of patients even experienced prolonged responses and improvement in Child-Pugh score in some cases.^15,23^ Notably, the pooled risk of irAEs was lower in the Child-Pugh B group, although this could be attributed to the shorter duration of therapy.

A recent meta-analysis assessed immunotherapy outcomes for patients with HCC and liver dysfunction but lacked the breadth of our systematic literature search and omitted studies involving immunotherapy plus locoregional treatments or tyrosine kinase inhibitors.^40^ Our study extends this work by acquiring patient data from 7 primary studies,^23,24,26,27,30,33,35^ which enabled comprehensive subgroup analyses that consider efficacy outcomes (ie, ORR, DCR, OS, and PFS) and safety profiles, screening more potential studies, and excluding overlapping cohorts.

A meta-analysis of sorafenib use in patients with Child-Pugh B advanced HCC showed a 4.2% response rate.^10^ However, recent investigations into ICIs, either alone or combined with tyrosine kinase inhibitors, showed promising clinical efficacy, manageable toxic effects, and favorable safety in patients with advanced HCC and Child-Pugh B liver function.^26,32,35^ Although indirect comparisons should be interpreted with caution, the response rate observed in our study was 14%, suggesting a potential therapeutic benefit from ICI therapy in a relevant proportion of these patients.

Additionally, the median OS reported for sorafenib therapy in the previous meta-analysis was 4.6 months for patients with Child-Pugh B HCC,^10^ and in some retrospective or prospective studies, the median OS was approximately 3 to 5 months.^8,41,42^ Notably, our research revealed that with ICI treatment, the median OS was 5.5 months. Although a median OS of less than 6 months still requires much work to improve the efficacy of our treatment options, this finding denotes an improvement over the median OS following sorafenib therapy. However, our findings suggest that in patients with advanced HCC and Child-Pugh B liver function, the efficacy of immunotherapy may be lower compared with Child-Pugh A despite competing comorbidity and a much higher risk of death from liver failure and other cirrhosis-related complications independent of cancer. Results were similar between ICI monotherapy and combination treatments (ie, ICIs combined with targeted therapy) separately.

Advanced liver dysfunction was postulated to polarize the liver microenvironment toward a more profound immunosuppression,^43^ implying reduced responsiveness to ICIs. Combination therapy, such as transcatheter arterial chemoembolization, radiofrequency ablation, and radiation therapy, was used in some studies.^15,20,25,29,36^ Since better liver function is an important factor for maximizing the therapeutic outcome of systemic therapy,^44^ the synergistic effect of ICIs combined with other therapies may be more pronounced in patients with Child-Pugh A liver function, which may have contributed to the significant difference in ORR and DCR between the 2 groups in our overall meta-analysis. Additionally, the smaller number of patients in the Child-Pugh B group, shorter follow-up and survival times, and different baseline characteristics between the Child-Pugh B and A groups may have contributed to the observed differences in response rates.

Regarding OS and PFS outcomes, among the 6 studies in the meta-analysis reporting a multivariable comparison of the Child-Pugh status,^21,26,27,28,37,39^ the pooled adjusted HR showed that Child-Pugh B liver function was associated with significantly worse OS. The unadjusted HR also indicated a reduced PFS for Child-Pugh B. Overall, ICI therapy was associated with a worse prognosis in patients with Child-Pugh B vs Child-Pugh A advanced HCC. Similar results were observed in subgroups of patients treated with ICI monotherapy and in combination with targeted therapies. However, because of the lack of an untreated Child-Pugh B control group, solid conclusions regarding a potential benefit of ICIs cannot be made. In fact, the prognosis of advanced HCC is determined not only by tumor burden but also by liver function.^45,46^ Our results confirm that liver function is associated with survival of patients with advanced HCC treated with ICIs. In patients with HCC, liver function is, in most cases, compromised due to the underlying liver fibrosis or cirrhosis but occasionally may also be impaired due to a high tumor burden. In patients with high tumor burden, response to treatment may stabilize or even improve liver function. Indeed, approximately 10% of patients enrolled in cohort 5 of the CheckMate 040 study experienced a significant improvement in liver function that lasted 6 months or longer.^15^

Our study also affirmed the safety of ICIs in patients with Child-Pugh B advanced HCC, revealing no significant differences in incidence of trAEs between the Child-Pugh A and B groups. This finding aligns with prior studies, including CheckMate 040,^15^ Ng et al,^20^ and Fessas et al,^26^ that have shown comparable safety between the groups. Interestingly, we observed a lower risk of irAEs for patients with Child-Pugh B, possibly due to their shorter exposure to ICIs because of shorter survival. Overall, our study suggests that ICIs, even in patients with advanced HCC with poor liver function, present manageable adverse effects and are safe. Notably, further subgroup analyses did not reveal any significant differences in radiologic response, survival, or incidence of trAEs between patients with CTP7 and CTP8/9 scores. However, due to the limited sample size (fewer than 100 cases with CTP8/9 across 6 studies^23,24,26,27,33,35^), further multicenter prospective cohort studies are needed to evaluate the association of CTP score with the efficacy and tolerability of ICI treatment in these patients.

### Limitations

Several limitations should be acknowledged. First, the majority of the included studies were retrospective, which may be subject to selection bias, and larger randomized clinical trials are recommended. Second, the smaller number of patients with Child-Pugh B liver function and baseline differences between the Child-Pugh B and A cohorts may represent potential sources of bias. However, the pooled adjusted HR was similar to the pooled unadjusted HR. Third, the confounding factors analyzed in multivariable Cox proportional hazards regression models varied across studies, although the pooled adjusted HR in the overall patient cohort showed low heterogeneity. Fourth, there may be potential underreporting of AEs in some studies, and the lack of an untreated Child-Pugh B cohort prevents definite conclusions regarding a potential survival benefit of ICI therapy in these patients. Fifth, we noticed high heterogeneity in some of our results, particularly in single-arm studies. Our attempts to detect potential sources of heterogeneity through meta-regression analyses did not yield any results. The high heterogeneity observed in the single-group meta-analysis may be attributable to the combination of single-group and comparative research data. Therefore, these results should be interpreted with caution. Sixth, 5 studies did not provide detailed information on the specific ICI agents used,^20,30,36,37,38^ which may limit the relevance of the data from these studies and highlights the importance of reporting detailed information on the ICI agents used in future studies.

## Conclusions

The findings of our systematic review and meta-analysis show that ICI therapy in patients with Child-Pugh B advanced HCC appears to be safe and associated with a significant number of radiologic responses, even though survival is inherently lower than in patients with Child-Pugh A HCC. Our data support the use of immunotherapy in well-selected patients with HCC and Child-Pugh B liver function, but randomized studies are needed to confirm the outcomes of ICI treatment in patients with advanced liver disease.

## Notes

Supplement 1. **eFigure 1.** Flowchart of Study Selection **eFigure 2.** Subgroup Analyses Based on the ICI Regimens by Single-Arm Meta-Analysis **eFigure 3.** Comparative Efficacy of ICI Treatment According to ORR and DCR Between Advanced HCC With Child-Pugh A and Child-Pugh B, Categorized by ICI Monotherapy and ICIs Combined With Targeted Therapy **eFigure 4.** Subgroup analysis based on the CTP Score in Child-Pugh B Group. **eFigure 5.** Example of Comparison of the Original Curves and the Reconstructed Curves of D’Alessio (2022) **eFigure 6.** Subgroup Analyses of Hazard Ratio of Overall Survival Based on the ICI Regimens **eFigure 7.** Sensitivity Analysis of the Main Outcomes **eFigure 8.** Funnel Plot for Assessing Potential Publication Bias of the 2 Main Outcomes **eTable 1.** Summary of Included Studies **eTable 2.** Quality Assessment of the Included Cohort Studies Using the Newcastle-Ottawa Scale **eTable 3.** The Case Series Report Quality Evaluation Form for the Included Single-Arm Studies **eTable 4.** Meta-Regression of Incidence of trAEs Among Patients With Child-Pugh B HCC vs Child-Pugh A HCC **eTable 5.** Meta-Regression of mOS in Patients With Advanced HCC With Child-Pugh B Liver Function **eAppendix 1.** Detailed Search Strategy **eAppendix 2.** Inclusion and Exclusion Criteria for Study Selection **eAppendix 3.** Supporting PRISMA Checklist Items

Supplement 2.
Data Sharing Statement
