# Supplementary material for: Immune Checkpoint Inhibitors for Child-Pugh Class B Advanced Hepatocellular Carcinoma: A Systematic Review and Meta-Analysis
Source: JAMA Oncol. 2023 Aug 24;9(10):1423–31. doi: 10.1001/jamaoncol.2023.3284 (PMC10450588; doi:10.1001/jamaoncol.2023.3284)
Supplement: Supplement 1. — eFigure 1. Flowchart of Study Selection eFigure 2. Subgroup Analyses Based on the ICI Regimens by Single-Arm Meta-Analysis eFigure 3. Comparative Efficacy of ICI Treatment According to ORR and DCR Between Advanced HCC With Child-Pugh A and Child-Pugh B, Categorized by ICI Monotherapy and ICIs Combined With Targeted Therapy eFigure 4. Subgroup analysis based on the CTP Score in Child-Pugh B Group. eFigure 5. Example of Comparison of the Original Curves and the Reconstructed Curves of D’Alessio (2022) eFigure 6. Subgroup Analyses of Hazard Ratio of Overall Survival Based on the ICI Regimens eFigure 7. Sensitivity Analysis of the Main Outcomes eFigure 8. Funnel Plot for Assessing Potential Publication Bias of the 2 Main Outcomes eTable 1. Summary of Included Studies eTable 2. Quality Assessment of the Included Cohort Studies Using the Newcastle-Ottawa Scale eTable 3. The Case Series Report Quality Evaluation Form for the Included Single-Arm Studies eTable 4. Meta-Regression of Incidence of trAEs Among Patients With Child-Pugh B HCC vs Child-Pugh A HCC eTable 5. Meta-Regression of mOS in Patients With Advanced HCC With Child-Pugh B Liver Function eAppendix 1. Detailed Search Strategy eAppendix 2. Inclusion and Exclusion Criteria for Study Selection eAppendix 3. Supporting PRISMA Checklist Items [file jamaoncol-e233284-s001.pdf]

Supplemental Online Content

Xie E, Yeo YH, Scheiner B, et al. Outcomes and tolerablity of immune checkpoint inhibitors for advanced hepatocellular carcinoma with Child-Pugh Class B liver function: a meta-analysis. *JAMA Oncol*. Published online August 24, 2023. doi:10.1001/jamaoncol.2023.3284

eFigure 1. Flowchart of Study Selection

eFigure 2. Subgroup Analyses Based on the ICI Regimens by Single-Arm Meta-Analysis

eFigure 3. Comparative Efficacy of ICI Treatment According to ORR and DCR Between Advanced HCC With Child-Pugh A and Child-Pugh B, Categorized by ICI Monotherapy and ICIs Combined With Targeted Therapy

eFigure 4. Subgroup analysis based on the CTP Score in Child-Pugh B Group.

eFigure 5. Example of Comparison of the Original Curves and the Reconstructed Curves of D'Alessio (2022)

eFigure 6. Subgroup Analyses of Hazard Ratio of Overall Survival Based on the ICI Regimens

eFigure 7. Sensitivity Analysis of the Main Outcomes

eFigure 8. Funnel Plot for Assessing Potential Publication Bias of the 2 Main Outcomes

eTable 1. Summary of Included Studies

eTable 2. Quality Assessment of the Included Cohort Studies Using the Newcastle-Ottawa Scale

eTable 3. The Case Series Report Quality Evaluation Form for the Included Single-Arm Studies

eTable 4. Meta-Regression of Incidence of trAEs Among Patients With Child-Pugh B HCC vs Child-Pugh A HCC

eTable 5. Meta-Regression of mOS in Patients With Advanced HCC With Child-Pugh B Liver Function

eAppendix 1. Detailed Search Strategy

eAppendix 2. Inclusion and Exclusion Criteria for Study Selection

eAppendix 3. Supporting PRISMA Checklist Items

This supplemental material has been provided by the authors to give readers additional information about their work.

© 2023 Xie E et al. *JAMA Oncology*.

eFigure 1. Flowchart of Study Selection

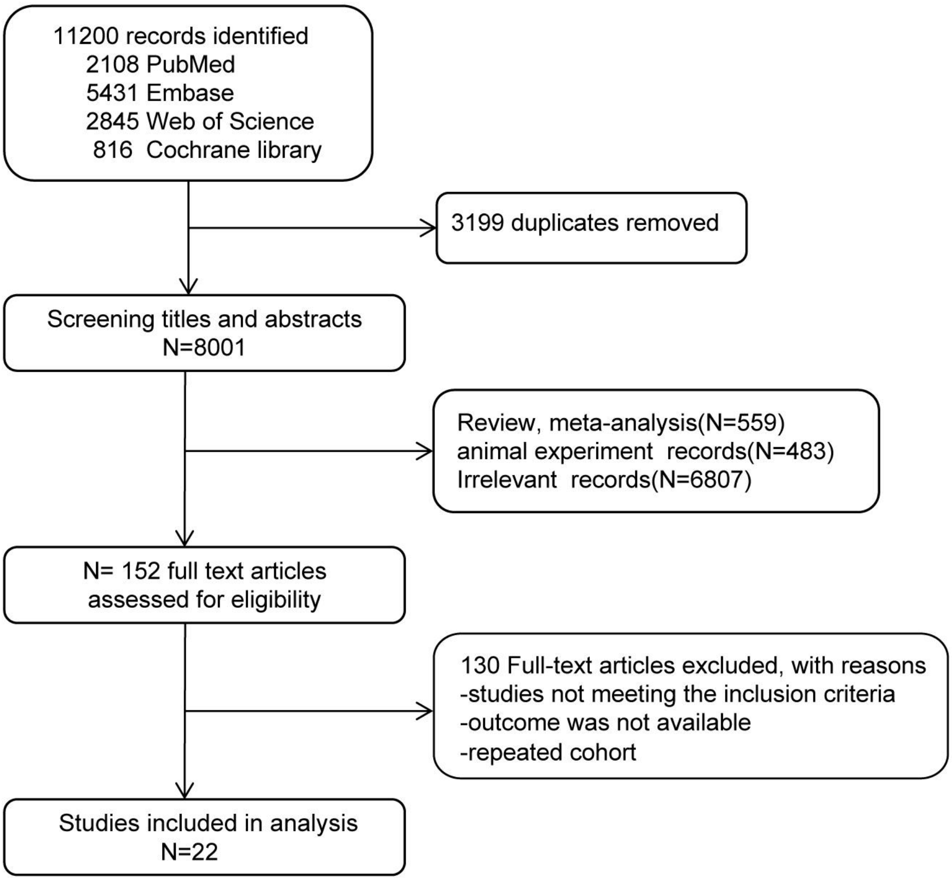

© 2023 Xie E et al. *JAMA Oncology*.

eFigure 2. Subgroup Analyses Based on the ICI Regimens by Single-Arm Meta-Analysis. (A) Objective response rate; (B) Disease control rate. ICI:Immune checkpoint inhibitor

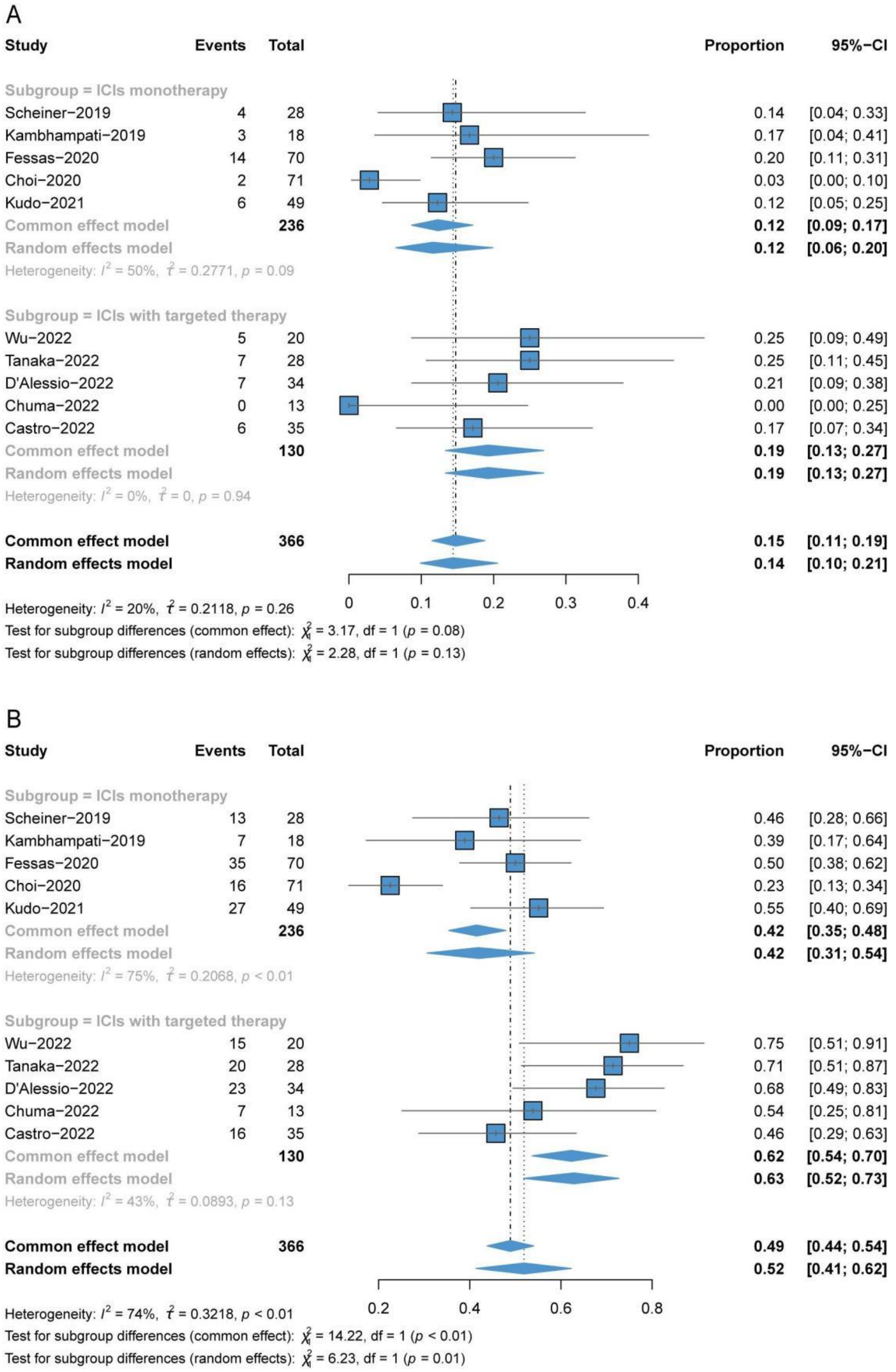

© 2023 Xie E et al. *JAMA Oncology*.

eFigure 3. Comparative Efficacy of ICI Treatment According to (A) ORR and (B) DCR Between Advanced HCC With Child-Pugh A and Child-Pugh B, Categorized by ICIs Monotherapy and ICIs Combined With Targeted Therapy. ICI:immune checkpoint inhibitor; ORR:objective response rate; DCR:disease control rate; HCC:hepatocellular carcinoma

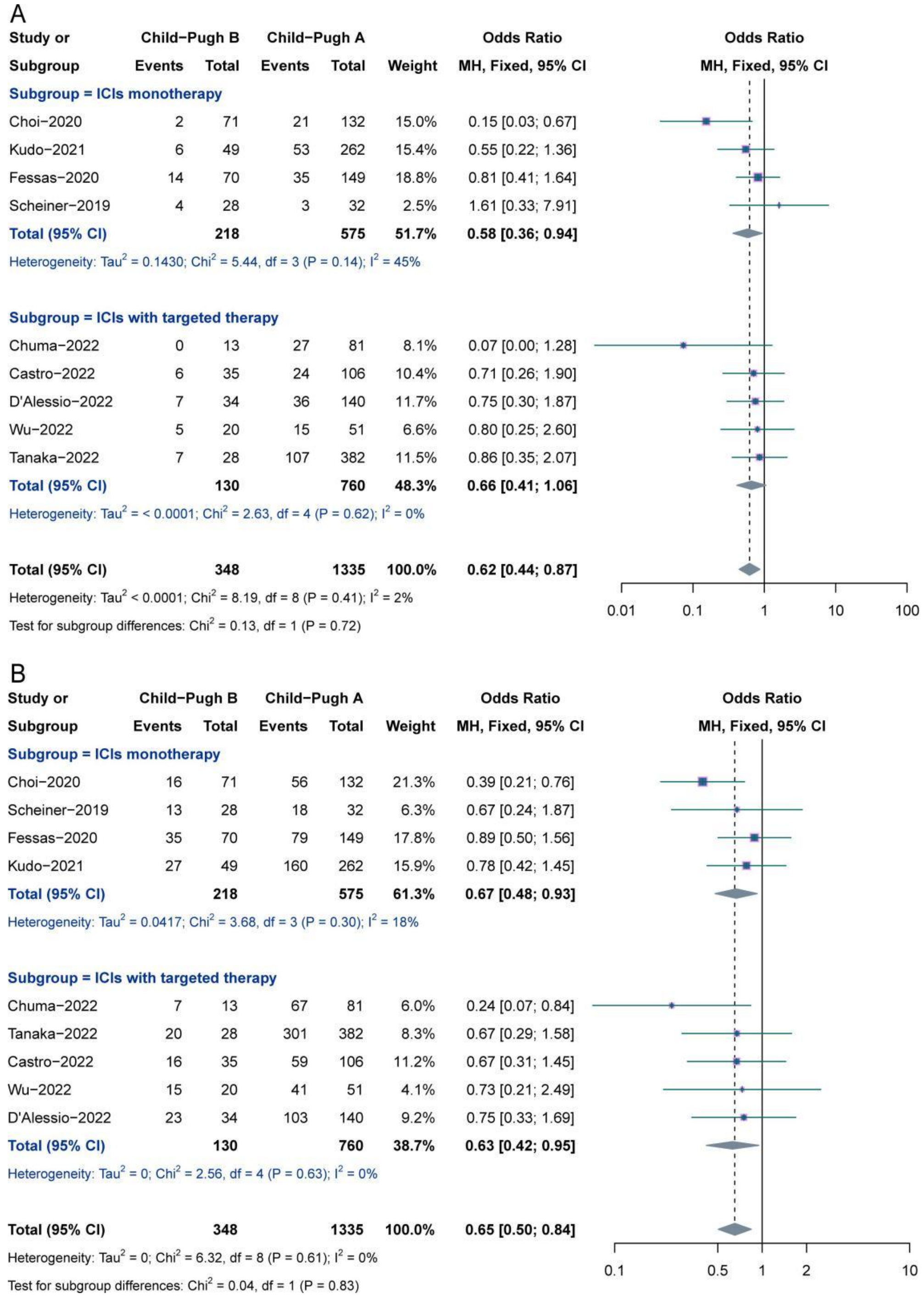

© 2023 Xie E et al. *JAMA Oncology*.

eFigure 4 Subgroup Analysis Based on the CTP Score in Child-Pugh B Group. (A-E) Assessing the differences in ORR, DCR, incidence of trAEs, OS, and PFS between the CTP8+9 and CTP7 separately. CTP:Child-Turcotte-Pugh; ORR: objective response rate; DCR: disease control rate; trAEs: treatment -related adverse events; OS: overall survival; PFS:progression-free survival; HR:hazard ratio

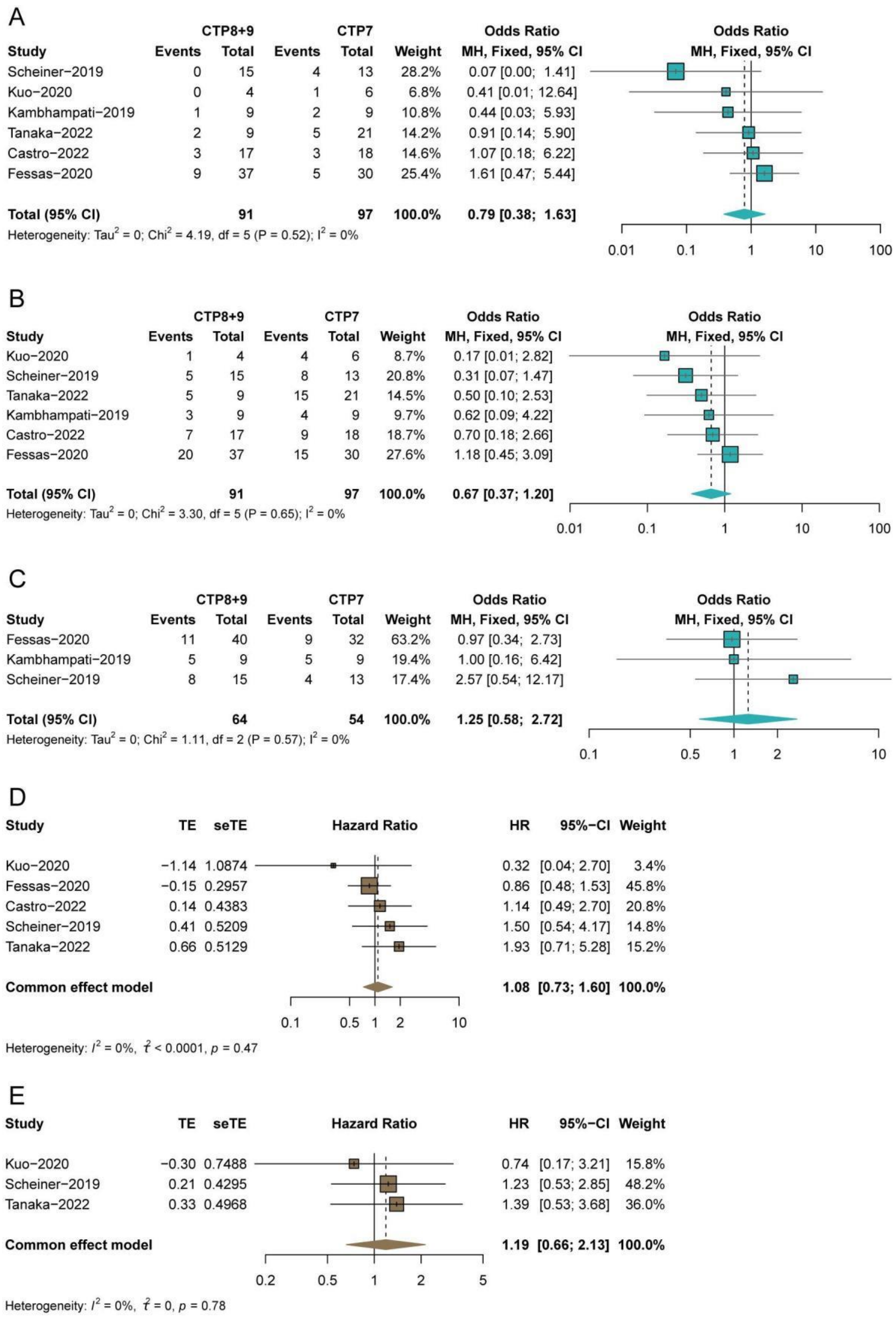

© 2023 Xie E et al. *JAMA Oncology*.

eFigure 5. Example of Comparison of the Original Curves and the Reconstructed Curves of D’Alessio (2022). CPA:Child-Pugh A; CPB:Child-Pugh B; OS:overall survival; PFS:progression-free survival

<table>
<tr><th>Original</th><th>Reconstructed</th></tr>
<tr><td>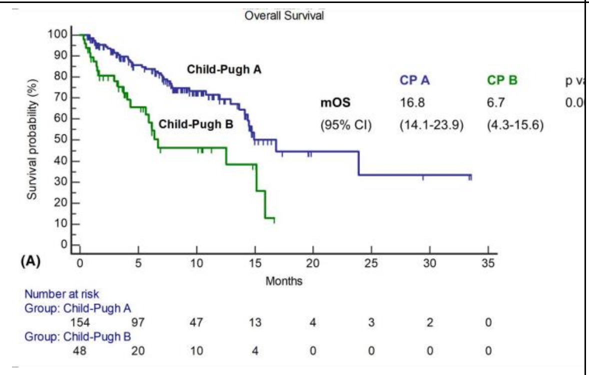
</td><td>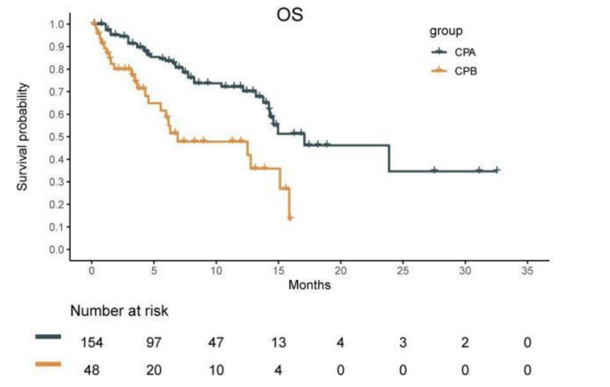
</td></tr>
<tr><td>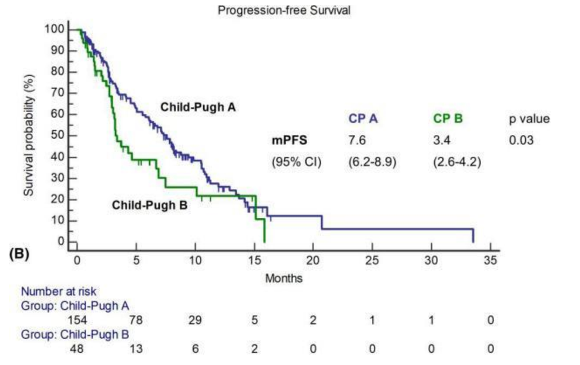
</td><td>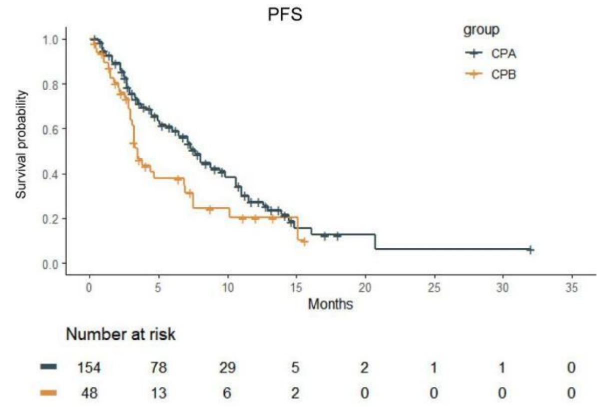
</td></tr>
</table>

© 2023 Xie E et al. *JAMA Oncology*.

eFigure 6. Subgroup Analyses of Hazard Ratio of Overall Survival Based on the ICI Regimens. ICI:Immune checkpoint inhibitor

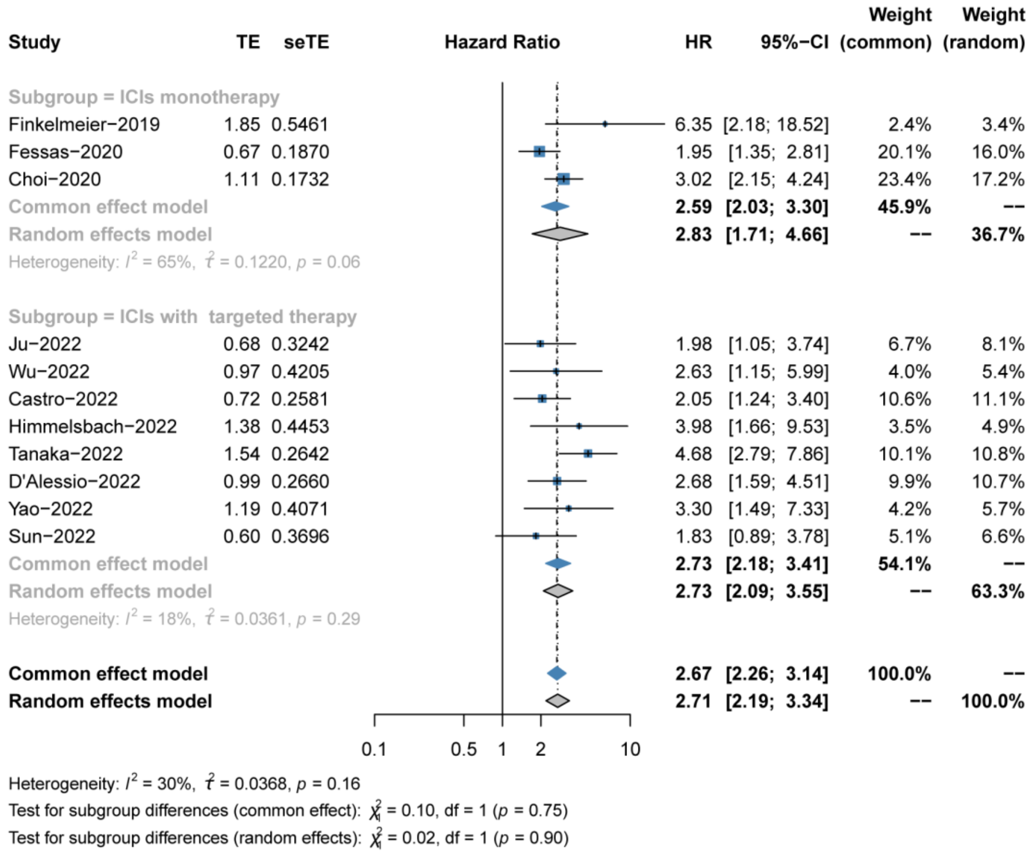

© 2023 Xie E et al. *JAMA Oncology*.

eFigure 7. Sensitivity Analysis of Main Outcomes. (A-C) Pooled odds ratio of objective response rate, hazard ratio of overall survival and odds ratio of treatment-related adverse events respectively; (D-F) Pooled objective response rates, incidence of treatment-related adverse events, median overall survival by single-arm meta-analysis.

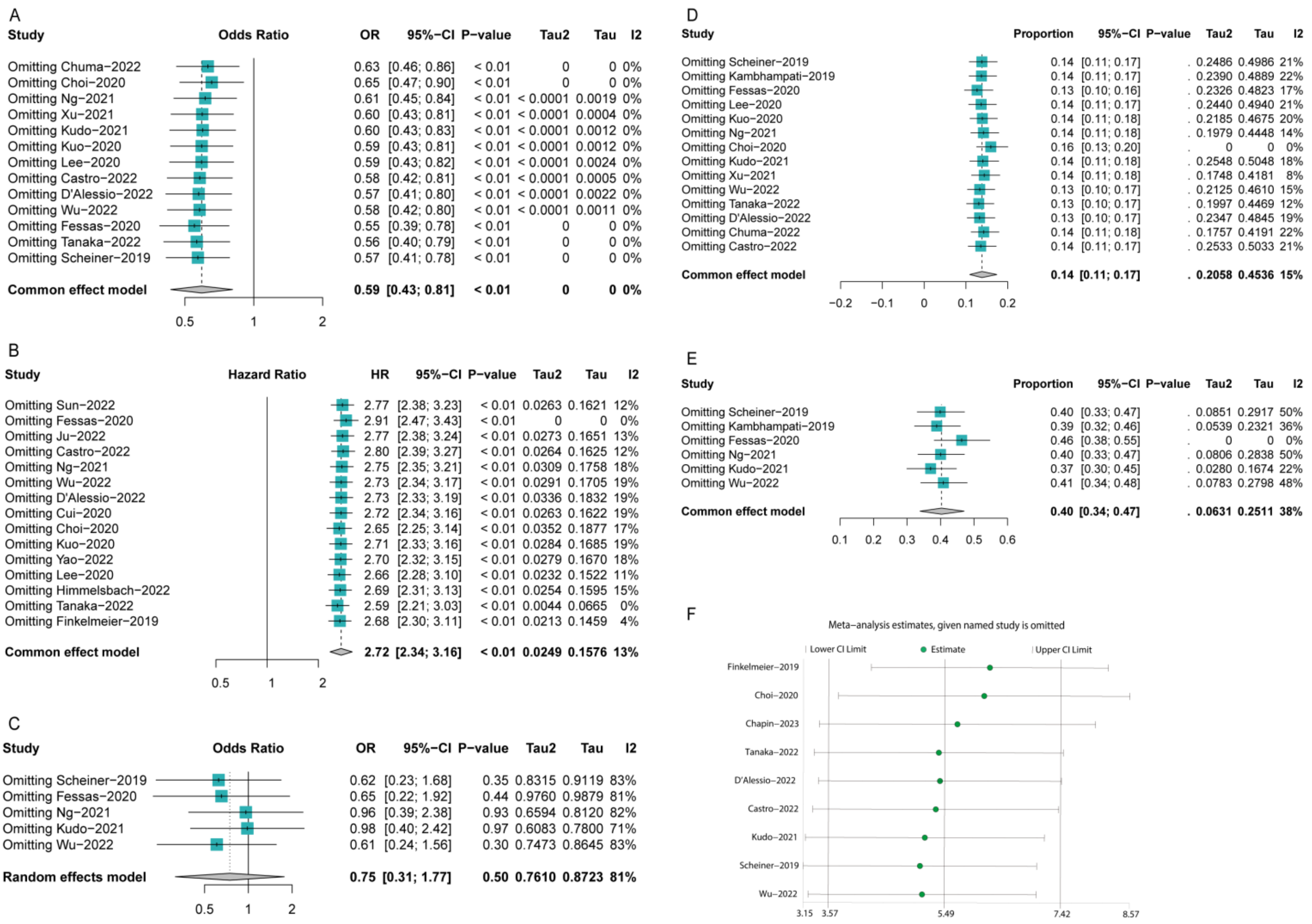

© 2023 Xie E et al. *JAMA Oncology*.

eFigure 8. Funnel Plot for Assessing Potential Publication Bias of the 2 Main Outcomes. (A) Pooled odds ratio of objective response rate. (B) Pooled hazard ratio of overall survival.

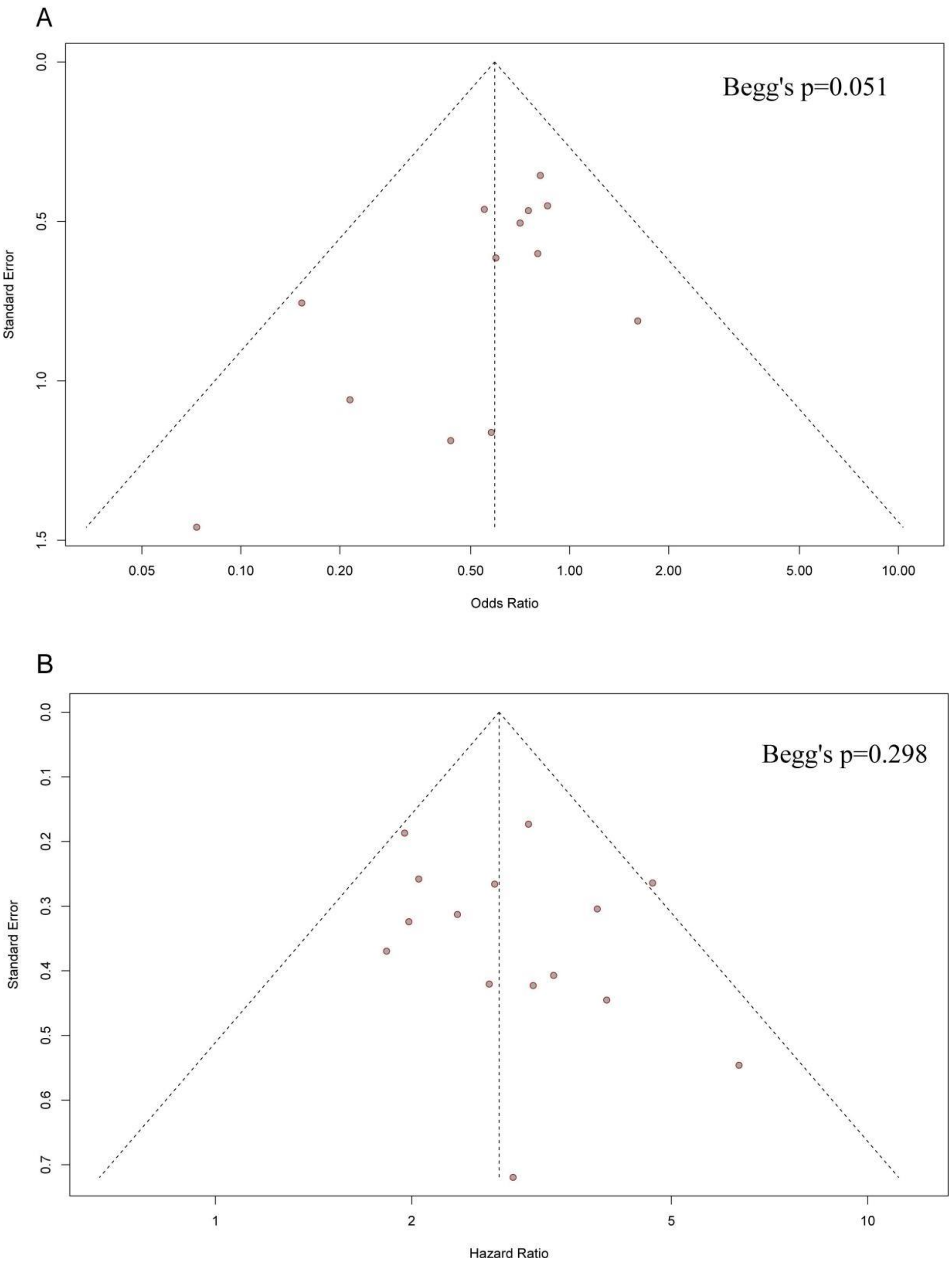

© 2023 Xie E et al. *JAMA Oncology*.

eTable 1. Summary of Included Studies

Author	Study design	ICI agent	Sample size	Child-Pugh No (%)	Mean±SD or median or range of age(years)	Extrahepatic metastases No (%)	Vascular invasion No (%)	Median follow-up (month)	BCLC No (%)		Etiologyn No(%)		
									A/B	C/D	HBV	HCV	Non -virus
Finkelmeier 2019	retrospective	Nivolumab	34	A 19 (56)<br>B 14 (41)	65(40–77）	19 (56)	19 (56)	3.3	5(15)	29 (85)	2 (6)	10 (29)	22(65)
Scheiner 2019	retrospective	nivolumab or pembrolizumab	65	A 32 (49)<br>B 28 (43)	65.2 ± 11.1	35(54)	24(37)	11.2	8 (12)	57(88)	8 (12)	10 (15)	47(72)
Kambhampati 2019	retrospective	Nivolumab	18	B 18(100)	66.5(26-86)	14 (78)	9(50)	NA	4 (22)	14 (78)	6 (33)	5 (28)	9(50)
Kuo 2020	retrospective	nivolumab or pembrolizumab	42	A 31 (76)<br>B 10 (24)	58	26(62)	24(57)	4.6	0 (0)	42 (100)	29 (78)	6 (16)	7(17)
Fessas 2020	prospective	Nivolumab	233	A 158 (68)<br>B 75 (32)	64 (56–69)	66(42)	59(38)	8	27(12)	206(88)	83 (36)	95 (41)	63(27)
Cui 2020	retrospective	nivolumab or pembrolizumab	55	A 35(64)<br>B 18(15)	56(40 - 83)	34(62)	22(40)	13	NA	NA	43	2	10(18)
Lee 2020	retrospective	nivolumab or pembrolizumab	95	A 69(73)<br>B 23(24)	65.5(57.2–72.9)	48 (51)	51 (54)	5.2	20 (21)	75 (79)	62 (65)	21 (22)	12(13)
Ng 2021	retrospective	ICIs	114	A 93（82）<br>B 21（18）	66(23–85)	86 (75)	58 (51)	13.8	6 (5)	108(95)	62 (54)	13 (11)	60(53)
Choi 2020	retrospective	nivolumab	203	A 132(65)<br>B 71(35)	56.9 ± 11.2<br>56.0 ± 9.4	120 (90.9)<br>64 (90.1)	46 (35)<br>42 (59)	5.6	6(5)<br>2(3)	126(96)<br>69(97)	111(84)<br>57(80)	4(3)<br>4(6)	17(13)<br>10(14)
Xu 2021	retrospective	PD-1	65	A 33 (51)<br>B 24 (37)	65	NA	NA	NA	NA	NA	57 (88)	NA	NA

© 2023 Xie E et al. *JAMA Oncology*.

eTable 1. Summary of Included Studies (continued)

Kudo 2021	prospective	nivolumab	311	A 262(84)<br>B 49(16)	63 (19–83)<br>67(40–78)	178 (68)<br>20 (41)	82 (31）<br>14 (29)	30<br>16.3	27(10)<br>10(20)	234 (89)<br>39(80)	66 (25)<br>8 (16)	60 (23)<br>21 (43)	136(52)<br>20(41)
Ju 2022	retrospective	camrelizumab	108	A 84(78)<br>B 24(22)	54(25-79)	NA	NA	13.5	18(17)	90(83)	92(85)	8(7)	8(7)
Wu 2022	prospective	pembrolizumab	71	A 51 (72)<br>B 20(28)	63 (28–89)	32 (45)	41 (58)	9.3	13 (18)	58(82)	45 (63)	11(16)	18(25)
Castro 2022	retrospective	atezolizumab	147	A 106 (72)<br>B 35 (24)	68.7 (30–96)	65 (45)	48 (33)	6.2	24(16)	123(84)	12 (8)	38 (26)	97(66)
Chapin 2023	retrospective	nivolumab	79	B 79(100)	66.6	26 (33)	27 (34)	NA	NA	NA	2 (3)	24 (30)	25(32)
Himmelsbach 2022	retrospective	Atezolizumab	66	A 35 (53)<br>B 23 (35)	65 (30–88)	NA	NA	7	23(35)	43(65)	9 (14)	14 (21)	43(65)
Tanaka 2022	retrospective	atezolizumab	457	A 427(93)<br>B 30(7)	74<br>74	140 (33)<br>14 (47)	76 (18)<br>9 (30)	8.7	183(43)<br>10(33)	237(56)<br>20(67)	78(17)<br>1(0)	144(25)<br>12(3)	204(48)<br>17(57)
D'Alessio 2022	retrospective	Atezolizumab	202	A 154 (76)<br>B 48 (24)	69 (23–90)	77 (38)	NA	9	58(29)	144 (71)	35 (17)	72 (36)	92(46)
Yao 2022	retrospective	PD-1	136	A 109 (80)<br>B 27 (20)	58 (14–84)	101 (74)	68 (50)	14.2	12 (9）	124 (91)	124 (91)	0 (0)	12 (9)
Ng_Tan 2021	retrospective	ICIs	168	A 131(78)<br>B 37(22)	69	110 (66)	84 (50)	25.1	28 (17)	140 (83)	97 (58)	15 (9)	56 (33)
Chuma 2022	retrospective	atezolizumab	94	A 81(86)<br>B 13(14)	72 (37–87)<br>73 (54–84)	27 (33)<br>5 (39)	20 (25)<br>2 (15)	4.8	41(51)<br>5(38)	40(49)<br>8(62)	18(22)<br>0	26(32)<br>5(38)	37(46)<br>8(6)
Sun 2022	retrospective	PD-1	84	A 72 (86)<br>B 12 (14)	53 (25–78)	42 (50)	NA	17.1	15 (18)	69 (82)	71 (85)	NA	NA

ICIs: Immune checkpoint inhibitors;BCLC: Barcelona clinic liver cancer classifification; HBV: Hepatitis B Virus; HCV: Hepatitis C Virus;PD-1: programmed cell death 1;NA:Not Available

© 2023 Xie E et al. *JAMA Oncology*.

eTable 2. Quality Assessment of the Included Cohort Studies Using the Newcastle-Ottawa Scale

Author	Year	Selection	Comparability	Outcome	Score
Choi	2020	4	1	3	8
Xu	2021	4	1	3	8
Ju	2022	4	2	2	8
Castro	2022	4	1	3	8
Chapin	2023	4	1	3	8
Tanaka	2022	4	1	2	7
Ng_Tan	2021	4	1	2	7
Chuma	2022	4	2	2	8
Sun	2022	4	1	3	8

© 2023 Xie E et al. *JAMA Oncology*.

eTable 3. The Case Series Report Quality Evaluation Form for the Included Single-Arm Studies

Study	Study objective	Study design	Study population	Intervention and co-intervention	Outcome measure	Statistical analysis	Results and conclusions	Competing interests and sources of support	Score
Finkelmeier 2019	1	2	3	1	3	1	4	1	16
Scheiner 2019	1	2	3	1	3	1	5	1	17
Kambhampati2019	1	1	3	1	3	1	4	1	15
Kuo 2020	1	1	3	2	3	1	3	1	15
Fessas 2020	1	3	3	2	3	1	4	1	18
Cui 2020	1	1	3	2	3	1	4	1	16
Lee 2020	1	1	3	2	3	1	5	1	17
Ng 2021	1	1	3	2	3	1	5	1	17
Kudo 2021	1	3	3	2	3	1	5	1	19
Wu 2022	1	2	3	2	3	1	4	1	17
Himmelsbach2022	1	2	3	2	3	1	5	1	18
D'Alessio 2022	1	2	3	2	3	1	4	1	17
Yao 2022	1	2	3	2	3	1	5	1	18

© 2023 Xie E et al. *JAMA Oncology*.

eTable 4. Meta-Regression of Incidence of trAEs Among Patients With Child-Pugh B HCC vs Child-Pugh A HCC

Variables	Coefficient	SE	*Z* value	*P* value	CI-Lower	CI-Upper
Year of publication	-0.0126	0.5098	-0.0247	0.9803	-1.0119	0.9867
Study design	-0.2887	1.0358	-0.2787	0.7805	-2.3188	1.7415
Sample size	-0.0045	0.0045	-1.0076	0.3136	-0.0133	0.0043
Proportion of non-virus	-1.4175	2.8079	-0.5048	0.6137	-6.9209	4.0859
Proportion of BCLC C/D	-15.9952	9.5391	-1.6768	0.0936	-34.6916	2.7012
Median follow-up	-0.2705	0.1661	-1.6285	0.1034	-0.596	0.0551
ICI regimens	-0.4052	0.5999	-0.6755	0.4994	-1.581	0.7706
Average age	-0.6413	0.352	-1.8219	0.0685	-1.3312	0.0486

eTable 5. Meta-Regression of mOS in Patients With Advanced HCC With Child-Pugh B Liver Function

Variables	Coefficient	SE	*Z* value	*P* value	CI-Lower	CI-Upper
Year of publication	-0.2554	0.2083	-1.2300	0.3450	-1.1516	0.6408
Study design	-0.8090	0.2817	-2.8700	0.1030	-2.0210	0.4031
Sample size	-0.0019	0.0010	-1.9800	0.1860	-0.0061	0.0022
Proportion of non-virus	-0.3109	1.2004	-0.2600	0.8200	-5.4759	4.8540
Proportion of BCLC C/D	-1.0603	1.3304	-0.8000	0.4620	-4.4802	2.3596
Median follow-up	0.0668	0.0491	1.3600	0.2320	-0.0594	0.1930
ICI regimens	0.3546	0.2615	1.3600	0.3080	-0.7704	1.4795
Average age	0.0903	0.0578	1.5600	0.2590	-0.1586	0.3391

© 2023 Xie E et al. *JAMA Oncology*.

eAppendix 1. Detailed Search Strategy

PubMed, up to June 15, 2022 (2,108 Articles)

(((((Hepatocellular carcinoma*[tw] OR Liver Cancer*[tw] OR Hepatoma*[tw] OR Liver Cell Carcinoma*[tw] OR Liver Neoplasm*[tw] OR Cancer of the liver[tw])) OR (("Carcinoma, Hepatocellular"[Mesh]) OR "Liver Neoplasms"[Mesh:NoExp])))) AND ((atezolizumab[Title/Abstract]) OR (avelumab[Title/Abstract]) OR (camrelizumab[Title/Abstract]) OR (cemiplimab[Title/Abstract]) OR (CTLA-4[Title/Abstract]) OR (cytotoxic T lymphocyte-associated protein 4[Title/Abstract]) OR (durvalumab[Title/Abstract]) OR (ICIs[Title/Abstract]) OR (immune checkpoint inhibitor[Title/Abstract]) OR (ipilimumab[Title/Abstract]) OR (nivolumab[Title/Abstract]) OR (PD-1[Title/Abstract]) OR (pembrolizumab[Title/Abstract]) OR (PD-L1[Title/Abstract]) OR (programmed cell death 1[Title/Abstract]) OR (programmed cell death ligand 1[Title/Abstract]) OR (sintilimab[Title/Abstract]) OR (toripalimab[Title/Abstract]) OR (tremelimumab[Title/Abstract]))

Embase, up to June 15, 2022 (5,431Articles)

#1 'atezolizumab':ti,ab OR 'avelumab':ti,ab OR 'camrelizumab':ti,ab OR 'cemiplimab':ti,ab OR 'ctla-4':ti,ab OR 'cytotoxic t lymphocyte-associated protein 4':ti,ab OR 'durvalumab':ti,ab OR 'icis':ti,ab OR 'immune checkpoint inhibitor':ti,ab OR 'ipilimumab':ti,ab OR 'nivolumab':ti,ab OR 'pd-1':ti,ab OR 'pd-l1':ti,ab OR 'pembrolizumab':ti,ab OR 'programmed cell death 1':ti,ab OR 'programmed cell death ligand 1':ti,ab OR 'sintilimab':ti,ab OR 'toripalimab':ti,ab OR 'tremelimumab':ti,ab

#2 'liver cell carcinoma'/exp OR 'liver tumor'/exp OR 'hepatocellular carcinoma*':ti,ab OR 'liver cancer*':ti,ab OR 'hepatoma*':ti,ab OR 'liver cell carcinoma*':ti,ab OR 'liver neoplasm':ti,ab OR 'cancer of the liver':ti,ab

#3 #1 AND#2

Web of Science Search up to June 15, 2022 (2,845 Articles)

1. TOPIC: (Liver Cell Carcinoma OR Liver Tumor OR Hepatocellcular Carcinoma*

OR Liver Cancer OR Hepatoma* OR Liver Cell Carcinoma* OR Liver Neoplasm

OR Cancer of the Liver) OR TITLE: (Liver Cell Carcinoma OR Liver Tumor OR

Hepatocellcular Carcinoma* OR Liver Cancer OR Hepatoma* OR Liver Cell

Carcinoma* OR Liver Neoplasm OR Cancer of the Liver)

2. TOPIC: atezolizumab OR avelumab OR camrelizumab OR cemiplimab OR CTLA-4 OR cytotoxic T lymphocyte-associated protein 4 OR durvalumab OR ICIs OR immune checkpoint inhibitor OR ipilimumab OR nivolumab OR PD-1 OR pembrolizumab OR PD-L1 OR programmed cell death 1 OR programmed cell death ligand 1 OR sintilimab OR toripalimab OR tremelimumab

3. 1 AND 2

Cochrane library, up to June 15, 2022 (816 Articles)

Title abstract keyword
Liver Cell Carcinoma OR Liver Tumor OR Liver Cancer OR Hepatocellular
Carcinoma OR Liver Neoplasm
AND
Title abstract keyword
atezolizumab OR avelumab OR camrelizumab OR cemiplimab OR CTLA-4 OR cytotoxic T lymphocyte-associated protein 4 OR durvalumab OR ICIs OR immune checkpoint inhibitor OR ipilimumab OR nivolumab OR PD-1 OR pembrolizumab OR PD-L1 OR programmed cell death 1 OR programmed cell death ligand 1 OR sintilimab OR toripalimab OR tremelimumab

© 2023 Xie E et al. *JAMA Oncology*.

eAppendix 2. Inclusion and Exclusion Criteria for Study Selection

Eligible studies were identified according to the following inclusion criteria:

(1) Participants: advanced HCC patients with Child-Pugh B liver function;

(2) Interventions: treatment with immune checkpoint inhibitors with or without TKIs and bevacizumab;

(3) Comparisons: advanced HCC patients with Child-Pugh A liver function;

(4) Outcomes: objective response rate (ORR, defined as patients with complete or partial response), disease control rate (DCR, defined as patients with complete response, partial response, or stable disease), progression-free survival (PFS, median, defined as the time from the date of first checkpoint inhibitor administration until radiological disease progression or death, whatever came first), overall survival (OS, median, defined as the time from the date of first checkpoint inhibitor administration until death), unadjusted or adjusted hazard ratio (HR) of OS or PFS and the corresponding 95% confidence interval (CI), incidence of treatment related adverse events (trAEs) and immunotherapy related adverse events (irAEs) assessed according to the National Cancer Institute's Common Terminology Criteria for Adverse Events (CTCAE).

(5) Study: randomized controlled trials (RCTs), cohort studies, or single-arm studies.

Upon identification of studies with overlapping patient cohorts, we only included data from the study with a more recent data cut-off, larger sample size, and/or more data available for subgroup analysis. Studies that met any of the following exclusion criteria were not included in analyses:

(1) repeated articles;

(2) animal experiments, case reports, review articles;

(3) diseases other than advanced HCC;

(4) studies without the aforementioned outcomes;

(5) analyses of fewer than 10 patients in either the Child-Pugh A or B cohort.

© 2023 Xie E et al. *JAMA Oncology*.

eAppendix 3. Supporting PRISMA Checklist Items

Section/topic	#	Checklist item	Reported on page #
**TITLE**			
Title	1	Identify the report as a systematic review, meta-analysis, or both.	1
**ABSTRACT**			
Structured summary	2	Provide a structured summary including, as applicable: background; objectives; data sources; study eligibility criteria, participants, and interventions; study appraisal and synthesis methods; results; limitations; conclusions and implications of key findings; systematic review registration number.	1
**INTRODUCTION**			
Rationale	3	Describe the rationale for the review in the context of what is already known.	2
Objectives	4	Provide an explicit statement of questions being addressed with reference to participants, interventions, comparisons, outcomes, and study design (PICOS).	2
**METHODS**			
Protocol and registration	5	Indicate if a review protocol exists, if and where it can be accessed (e.g., Web address), and, if available, provide registration information including registration number.	2
Eligibility criteria	6	Specify study characteristics (e.g., PICOS, length of follow-up) and report characteristics (e.g., years considered, language, publication status) used as criteria for eligibility, giving rationale.	2, Appendix 2.
Information sources	7	Describe all information sources (e.g., databases with dates of coverage, contact with study authors to identify additional studies) in the search and date last searched.	2
Search	8	Present full electronic search strategy for at least one database, including any limits used, such that it could be repeated.	2, Appendix 1
Study selection	9	State the process for selecting studies (i.e., screening, eligibility, included in systematic review, and, if applicable, included in the meta-analysis).	2, eFig.1

© 2023 Xie E et al. *JAMA Oncology*.

Data collection process	10	Describe method of data extraction from reports (e.g., piloted forms, independently, in duplicate) and any processes for obtaining and confirming data from investigators.	2
Data items	11	List and define all variables for which data were sought (e.g., PICOS, funding sources) and any assumptions and simplifications made.	2, Appendix 1
Risk of bias in individual studies	12	Describe methods used for assessing risk of bias of individual studies (including specification of whether this was done at the study or outcome level), and how this information is to be used in any data synthesis.	2
Summary measures	13	State the principal summary measures (e.g., risk ratio, difference in means).	2
Synthesis of results	14	Describe the methods of handling data and combining results of studies, if done, including measures of consistency (e.g., I^2) for each meta-analysis.	2-3
Risk of bias across studies	15	Specify any assessment of risk of bias that may affect the cumulative evidence (e.g., publication bias, selective reporting within studies).	3
Additional analyses	16	Describe methods of additional analyses (e.g., sensitivity or subgroup analyses, meta-regression), if done, indicating which were pre-specified.	3
**RESULTS**			
Study selection	17	Give numbers of studies screened, assessed for eligibility, and included in the review, with reasons for exclusions at each stage, ideally with a flow diagram.	3, eFig.1
Study characteristics	18	For each study, present characteristics for which data were extracted (e.g., study size, PICOS, follow-up period) and provide the citations.	3, eTab.1
Risk of bias within studies	19	Present data on risk of bias of each study and, if available, any outcome level assessment (see item 12).	3
Results of individual studies	20	For all outcomes considered (benefits or harms), present, for each study: (a) simple summary data for each intervention group (b) effect estimates and confidence intervals, ideally with a forest plot.	3, eTab.1
Synthesis of results	21	Present results of each meta-analysis done, including confidence intervals and measures of consistency.	3-6
Risk of bias across studies	22	Present results of any assessment of risk of bias across studies (see Item 15).	6
Additional analysis	23	Give results of additional analyses, if done (e.g., sensitivity or subgroup analyses, meta-regression [see Item 16]).	5-6
**DISCUSSION**			

© 2023 Xie E et al. *JAMA Oncology*.

Summary of evidence	24	Summarize the main findings including the strength of evidence for each main outcome; consider their relevance to key groups (e.g., healthcare providers, users, and policy makers).	6-7
Limitations	25	Discuss limitations at study and outcome level (e.g., risk of bias), and at review-level (e.g., incomplete retrieval of identified research, reporting bias).	7-8
Conclusions	26	Provide a general interpretation of the results in the context of other evidence, and implications for future research.	8
**FUNDING**			
Funding	27	Describe sources of funding for the systematic review and other support (e.g., supply of data); role of funders for the systematic review.	8

© 2023 Xie E et al. *JAMA Oncology*.
