# Supplementary material for: Immune Checkpoint Inhibitors for Child-Pugh Class B Advanced Hepatocellular Carcinoma: A Systematic Review and Meta-Analysis
Source: JAMA Oncol. 2023 Aug 24;9(10):1423–31. doi: 10.1001/jamaoncol.2023.3284 (PMC10450588; doi:10.1001/jamaoncol.2023.3284)
Supplement: Supplement 2. — Data Sharing Statement [file jamaoncol-e233284-s002.pdf]

Data Sharing Statement

Xie. Immune Checkpoint Inhibitors for Child-Pugh Class B Advanced Hepatocellular Carcinoma. *JAMA Oncol*. Published August 24, 2023. doi:10.1001/jamaoncol.2023.3284

Data
Data available: No
